# 

**Published:** 1889-03

**Authors:** 


### CONTENTS:

Article I.—Headache from Eye Strain.
By Dr. Wheelock Rider. .......................... 481
" II.—History of Dentistry.......................... 493
" III.—Faith Healing. By Parsons Shaw, D.D.S ........... 501
" IV.—What Do You Know About Amalgam.............. 511
" V.—Chemistry, Physiology and Pathology of Nitrous
Oxide—Practical Points .......................... 515
" VI.—Diseases of the Maxillary Sinus or Antrum.
By Dr. Louis Jordan.............................. 518
Chicago Dental Society.......................................... 522

### EDITORIAL.

The Seventh Annual Commencement of the University of
Maryland Dental Department .......................... 523
Annual Commencement of the Baltimore College of Dental
Surgery ................................................ 526

